# Intestinal Giardiasis Disguised as Ulcerative Colitis

**DOI:** 10.1155/2018/8968976

**Published:** 2018-05-08

**Authors:** Yu Zhen, Lin Liao, Hu Zhang

**Affiliations:** ^1^Department of Gastroenterology, West China Hospital, Sichuan University, Chengdu, Sichuan Province, China; ^2^Department of Parasitology, College of Basic and Forensic Medicine, Sichuan University, Chengdu, Sichuan Province, China

## Abstract

Parasite-associated colitis is quite rare in clinical practice of Ulcerative Colitis (UC). Here we reported an intestinal giardiasis case that has been diagnosed with UC. Further examination of stool revealed cysts of* Giardia*. This case completely responded to Albendazole. Giardiasis should be included for the differential diagnosis of UC.

## 1. Introduction

Inflammatory Bowel Disease (IBD) comprises Ulcerative Colitis (UC) and Crohn's Disease (CD); the different progression and complex differential diagnosis of these two conditions thus make it difficult to diagnose IBD. Colitis can be attributed to various infections such as bacterial, viral, fungal, and protozoan. The conditions caused by pathogens like* Mycobacterium tuberculosis*,* Clostridium difficile*,* Cytomegalovirus* (CMV), EB virus (EBV), and occasionally Amoeba can masquerade as IBD [1–4], and vice versa. Moreover, the colon may develop a monomorphic appearance when attacked by different pathogenic agents [4], which makes it even harder to distinguish between IBD and non-IBD on the basis of endoscopy and histopathology. Furthermore, the widespread therapeutic use of corticosteroids, immunosuppressors (IMS), and biological agents has been associated with a higher risk of opportunistic infections which can disturb diagnosis and management of IBD [3, 5].

Giardiasis is one of the most common protozoan gastrointestinal diseases worldwide and is caused by* Giardia duodenalis (G. duodenalis)* [6].* G. duodenalis* is transmitted through the ingestion of cysts in contaminated food or water, or directly via the fecal/oral route [7]. The characteristic symptoms include abdominal cramps, nausea, acute or chronic diarrhea, malabsorption with weight loss, and failure to thrive in children when the intestine is infested [8, 9]. The life cycle of* G. duodenalis* consists of two stages—the replicative stage characterized by the trophozoites and the infectious stage which comprises cysts. Following ingestion, the cysts undergo excystation into trophozoites in the duodenum of small intestine due to the latter's acidic environment [10, 11]. The active trophocytes then divide mitotically and are eventually triggered to form environmentally resistant cysts, which pass into the large intestine and are excreted along with the feces [12]. These infectious cysts can survive for weeks to months in soil and water [11]. Giardiasis is routinely diagnosed by the microscopic examination of stools for the presence of cysts or trophozoites. Fecal examination further serves to confirm diagnosis in addition to the typical clinical features. It has been reported that in case of coexistent bacterial or viral infections,* G. duodenalis* infection can manifest as erosion or ulcerations of the intestinal mucosa as seen by endoscopy and can be difficult to distinguish from UC or CD. There are several antigiardial drugs, such as the current Albendazole (benzimidazole compounds), which is highly effective in treating giardiasis [13, 14].

We present a case of chronic giardiasis as documented by the detection of cysts of* G. duodenalis* in the stool specimen test and extensive hyperaemia, erosion, and superficial ulcerations under endoscopic examination, which was initially misdiagnosed as UC.

## 2. Case Study

**Figure 1:**
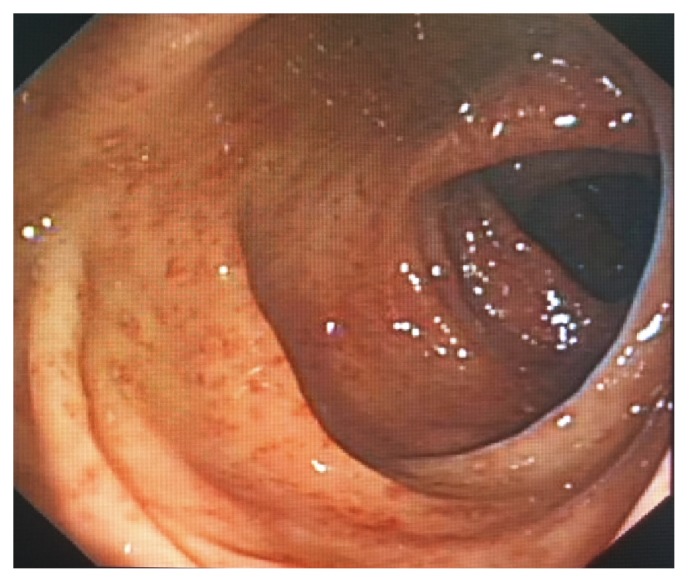
Diffused inflammation in the colon under coloscopy. The endoscopic images revealed that the inflammation of colonic mucosa is diffuse, continuous, and with extensive hyperaemia, erosion, and superficial ulcerations.

**Figure 2:**
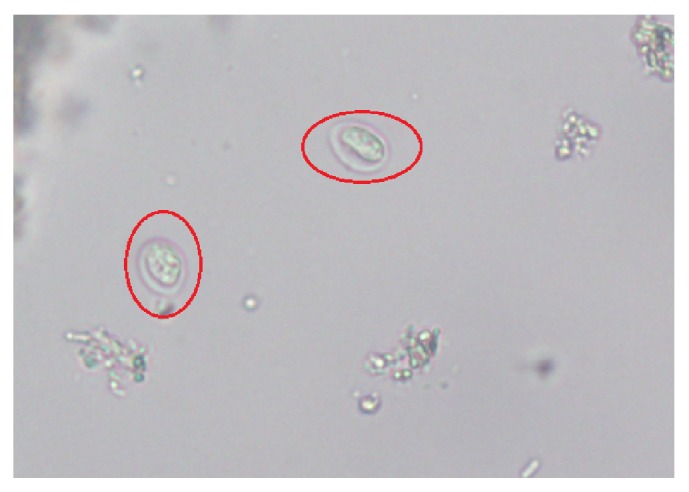
Stool examination showing cysts of* Giardia lamblia* (red circles).

A 61-year-old male suffering from chronic diarrhea and abdominal pain after eating contaminated food a year ago was referred to our unit. He was initially diagnosed with UC on the basis of endoscopic appearance at the local hospital. He was affected with persistent hypogastric cramps along with mucous stool. These symptoms underwent remission after oral administration of mesalazine. The aggravation of diarrheal symptoms started approximately three months before hospitalization and did not include fever, nausea, or vomiting. Routine blood test performed at the time of hospitalization showed normal levels of WBC at 4.15*∗*10^9^/L, hemoglobin at 128 g/L, and albumin at 37.0 g/L. The inflammatory marker C-reactive protein and erythrocyte sedimentation rate (ESR) also did not show any elevation. Serological examination detected elevated levels of immunoglobulins (IgG) against CMV (122 U/ml) and HSVI/II (22.7 Index). Physical examination showed a soft abdomen with normal bowel sound while a feces routine found a weakly positive result of occult test with no other significant abnormality. Endoscopy revealed terminal ileitis of focal hyperaemia and erosion, pancolitis of extensive dotted hyperaemia, erosion and superficial ulcerations with regular pouch, and no intestinal stenosis (Figure 1). Biopsy indicated mild to moderate chronic active inflammation, focal erosion of mucosa concomitant with granulation proliferation, infiltration of eosinophils, and lymphadenia. CTE detected a thickening of the wall of ileocecum and ascending colon with linear enhancement of mucosa. In order to exclude any potential infection of parasites before a definite diagnosis of UC could be made, his fresh stool was sent to the Department of Parasites in our home University. And the repeated stool examination revealed cysts of Giardia thus confirming giardiasis (Figure 2) and contaminated food was suspected to be the most likely source of the infection. Consequently, the patient was placed on oral Albendazole, a single dose of 400 mg per day for five days, and his symptoms substantially responded to the treatment, and he presented neither diarrhea nor abdominal pain one week later, and endoscopy performed one month after discharge showed significant improvement in the lesions of ileo-cecum and colon. We followed up this patient for about three years, and found no recurrence, and the stool examination revealed an absence of* G. duodenalis*. A definitive diagnosis of intestinal giardiasis was confirmed again.

## 3. Discussion

Definitive diagnosis of IBD is based primarily on the exclusion of other infectious diseases. Although giardiasis is usually not presented in the common differential diagnosis of IBD, in this case study, chronic giardiasis manifested as watery diarrhea and abdominal cramps which typically resembles UC. Giardiasis can be diagnosed by detection of cysts or trophozoites via microscopic stool examination [15], as well as by endoscopy and histopathology [16]. Since microscopic examination of a single stool sample cannot exclude* G. duodenalis* infection, at least three successive stool specimens should be examined, because of the intermittent nature of cyst excretion. Besides, cysts or trophozoites may be absent due to prior treatment with antibiotics, washing out of enema, or inappropriate collection and transportation; therefore, absence of cysts is also not a final confirmation of UC and CD. Whenever clinicians deal with patients with diarrhea, cramps, and nausea or children presenting abdominal pain and asthenia, especially with a history of consuming contaminated food or water and/or travel in endemic areas, they ought to take into consideration the possibility of parasitic infections by* G. duodenalis.* Repeated stool examination and multiple biopsies for cysts or trophozoites can be helpful in excluding giardiasis and other parasitic infections. Besides* G. duodenalis*, two other parasitic infections caused by either* E. histolytica or Cryptosporidium* spp. should also be taken into account.* E. histolytica* can cause amoebic dysentery, which presents with abdominal pain, diarrhea, or even bloody diarrhea. Its complications can include inflammation of the colon, perforation, or even peritonitis. Its diagnosis is based on the classical symptoms and detection of cysts or trophozoites stools under microscope. In contrast,* Cryptosporidium* spp. in gastrointestinal tract can result in cryptosporidiosis characteristic of watery diarrhea and vomiting, and so on. Cryptosporidiosis can be diagnosed based on the presence of* Cryptosporidium* in stool, but it is difficult to detect* Cryptosporidium *spp., so patients should submit several stool samples over several days. Stool specimens can be examined microscopically using different techniques (acid-fast staining, direct fluorescent antibody [DFA], etc.). As for the treatment of giardiasis, there are some medications for it. But, currently, Albendazole is the principle therapeutic drug for treating giardiasis with the advantages of fewer side effects and can result in total remission following regular treatment [13, 14].

It is known that the risk of opportunistic infections including HBV, CMV, EBV, tuberculosis, and* C. difficile* can be higher in IBD patients [3]. IBD and giardiasis can also coexist, as reported in a study of two CD cases with secretory diarrhea and concurrent giardiasis confirmed by the presence of* G. duodenalis* cysts in the patients' stools [17]. Considering the widespread use of antibiotics and the recent surge in IMS and corticosteroids for IBD treatment, it is necessary to draw sufficient attention to the prevalence of opportunistic infections in IBD patients.

In conclusion, a confirmation of IBD is incumbent on excluding other infectious diseases including giardiasis, which can mimic UC or CD. Remarkably, the absence of cysts or trophozoites cannot completely exclude giardiasis, making repeated stool examination and multiple biopsies essential to distinguishing between IBD and giardiasis.

## References

[1] Pai S. A. (2009). Amebic colitis can mimic tuberculosis and inflammatory bowel disease on endoscopy and biopsy.

[2] Chachu K. A., Osterman M. T. (2016). How to diagnose and treat IBD mimics in the refractory IBD patient who does not have IBD.

[3] Ng S. C., Chan F. K. (2013). Infections and inflammatory bowel disease: Challenges in Asia.

[4] Ibrahim T. M., Iheonunekwu N., Vantapool H. (2005). Differentiating amoebic ulcero-haemorrhagic recto-colitis from idiopathic inflammatory bowel disease: Still a diagnostic dilemma.

[5] Colombel J.-F., Loftus E. V., Tremaine W. J. (2004). The safety profile of infliximab in patients with Crohn's disease: the Mayo clinic experience in 500 patients.

[6] Minetti C., Chalmers R. M., Beeching N. J., Probert C., Lamden K. (2016). Giardiasis.

[7] Halliez M. C. M., Buret A. G. (2013). Extra-intestinal and long term consequences of Giardia duodenalis infections.

[8] Almirall P., Núñez F. A., Bello J., González O. M., Fernández R., Escobedo A. A. (2013). Abdominal pain and asthenia as common clinical features in hospitalized children for giardiasis.

[9] Bartelt L. A., Sartor R. B. (2015). Advances in understanding Giardia: Determinants and mechanisms of chronic sequelae.

[10] Ryan U., Cacciò S. M. (2013). Zoonotic potential of *Giardia*.

[11] Ankarklev J., Jerlström-Hultqvist J., Ringqvist E., Troell K., Svärd S. G. (2010). Behind the smile: Cell biology and disease mechanisms of Giardia species.

[12] Einarsson E., Ma'ayeh S., Svärd S. G. (2016). An up-date on Giardia and giardiasis.

[13] Granados C. E., Reveiz L., Uribe L. G., Criollo C. P. Drugs for treating giardiasis.

[14] Karabay O., Tamer A., Gunduz H., Kayas D., Arinc H., Celebi H. (2004). Albendazole versus metronidazole treatment of adult giardiasis: An open randomized clinical study.

[15] Koehler A. V., Jex A. R., Haydon S. R., Stevens M. A., Gasser R. B. (2014). Giardia/giardiasis - A perspective on diagnostic and analytical tools.

[16] Yakoob J., Jafri W., Abid S. (2005). Giardiasis in patients with dyspeptic symptoms.

[17] Desai T., Craig R. M. (1989). Secretory diarrhea in crohn's disease with concurrent giardiasis.

